# Small/Kiddie Cigarette Packaging Size and Its Impact on Smoking: A Systematic Review

**DOI:** 10.3390/ijerph191912051

**Published:** 2022-09-23

**Authors:** Halizah Mat Rifin, Miaw Yn Jane Ling, Tania Gayle Robert Lourdes, Thamil Arasu Saminathan, Wan Shakira Rodzlan Hasani, Nur Liana Ab Majid, Hamizatul Akmal Abd Hamid, Mohd Ruhaizie Riyadzi, Ahzairin Ahmad, Muhammad Fadhli Mohd Yusoff, Nor Asiah Muhamad

**Affiliations:** 1 Institute for Public Health, National Institutes of Health, Ministry of Health, Shah Alam 40170, Malaysia; 2 Sector for Evidence-Based Healthcare, National Institutes of Health, Ministry of Health, Shah Alam 40170, Malaysia

**Keywords:** smoking, kiddie packs, small packs, cigarette, packaging, size, mini packs

## Abstract

**Background:** Small cigarette pack sizes contain less than 20 cigarette sticks in a pack. Smaller packs may suggest lower costs, increasing affordability among lower-income users, especially the younger generation, which could lead to tobacco-related diseases and economic costs, including human capital lost results from tobacco-attributable morbidity and mortality. This concern has caused many countries to ban the sale of single cigarette sticks or kiddie packs. However, small cigarette pack sizes were proposed recently to be reintroduced by the tobacco industry with an excuse to prevent consumers from buying illicit cigarettes. This would demean efforts in combating tobacco consumption based on the existing tobacco control policies to prevent minors from purchasing cigarettes. Given the competing influences of affordability and availability of tobacco on consumption and the dearth of evidence-based review on the impact of pack size on smoking, this systematic review was conducted to identify the link between kiddie packs and smoking specifically on the initiation of smoking, urge/tendency to buy cigarettes among the general population and attempt to reduce cigarette consumption and prevalence of smoking using kiddie packs among current smokers. **Methods:** We include all studies except for reviews, guidelines, conference papers, commentaries, editorials, or opinion pieces. A database search was conducted in PubMed, EMBASE, CENTRAL, Web of Science and Scopus on 27 November 2021. The results were presented in the form of narrative synthesis under four groups: initiation of smoking; urge/tendency to buy cigarettes; the prevalence of smoking, and attempt to reduce cigarette consumption. The literature search identified 1601 articles, of which 21 articles had met the inclusion criteria. The methodological quality of all included articles was determined using a validated 16-item quality assessment tool (QATSDD). The average quality score for all papers was 34.8%. **Discussion:** Given the diverse study settings of the articles and despite the challenges of the methodological quality of some articles, this review provides some evidence that kiddie packs may increase the urge/tendency to buy cigarettes and mixed evidence on the attempt to reduce cigarette consumption. This review also found some evidence that kiddie pack purchasing among teenage smokers was higher compared to adults. However, we are uncertain about the link between kiddie packs and smoking initiation. Nevertheless, since most studies were of low quality, further high-quality studies are needed to conclude about the impact of kiddie packs on smoking to assist the policymakers and stakeholders in formulating new policies and strengthening existing strategies related to the kiddie packs.

## 1. Introduction

In the year 2015, over 1.1 billion people smoked tobacco globally. About six million people per year die (prematurely) due to tobacco use (smoked and smokeless), according to the World Health Organisation [1]. Smoked tobacco includes manufactured cigarettes, kretek and hand-rolled cigarettes and smokeless include snuff tobacco, electronic cigarette and chew tobacco [2]. Although there is a general awareness of the harmful effect of smoking, there are more than one billion users of these products worldwide [3]. According to the Word Health Organisation, over 80% of tobacco users live in low-and-middle-income countries, with the highest burden of tobacco-related illness and death. Tobacco use might lead to poverty as the user switches the household spending of basic needs to tobacco [4].

There are many ways cigarettes are sold, such as in tins, cartons, packs, “kiddie” packs, and loose sticks [5]. Small cigarette pack sizes (hereafter referred to as kiddie packs) consist of less than 20 cigarette sticks per pack [6]. It is sold in packages of 5, 10 or 15 cigarette sticks in many countries [7]. Kiddie packs are also known as ‘mini packs’ or ‘small packs’ [8]. Tobacco companies introduced kiddie packs into the market with the claim that they might support moderation and encourage smoking cessation among heavy smokers [9]. A study revealed that cigarette consumption per day was positively associated with pack size [10]. In addition, small cigarette packs are used by some smokers as a method of controlling their consumption [10]. Nevertheless, there is still a lack of experimental evidence for a causal relationship between pack size and consumption [10]. On the other hand, the affordability of kiddie packs would encourage smoking among the lower income groups, mainly teens and minors [5]. This would demean efforts in combating tobacco consumption, as numerous studies have shown that tobacco consumption dropped in response to higher prices [11,12,13]. In Indonesia, based on the 2014 Global Youth Tobacco Survey (GYTS), three out of five Indonesian students aged between 13 and 15 could buy cigarettes easily [14]. This finding was supported by another study conducted in Denpasar, Bali, which showed that more than half of retailers sold cigarettes in single sticks. Its buyers were mostly young people as it is within their buying capacity compared to buying a whole cigarette pack [15]. Besides, Indonesia, the Philippines and Thailand allowed cigarettes to be sold in single sticks and kiddie packs, thus making them easily accessible and attractive to teens and minors [16].

Most adult smokers started when they were teenagers [17]. According to a study in 2016, among 143 teenage smokers, 70% started smoking between 12 to 15 years old [17]. Near to 60% did not even enjoy smoking and attempted to quit without success [17]. Another study in Ireland among those aged eight years and above also revealed that 78% of smokers started smoking before they reached 18, and 53% before they got 15 [18]. This showed that smoking initiation is primarily a phenomenon affecting kids and teenagers [18]. This is quite worrisome as smokers would expose themselves to the harmful effect of cigarettes at an incredibly early age, leading to diseases caused by smoking [17]. The tobacco economic cost is significant and includes remarkable health care costs for treating conditions caused by tobacco use. In addition, there are also human capital losses resulting from tobacco-attributable morbidity and mortality [13]. Based on the above-stated points, the World Health Organisation (WHO) Framework Convention on Tobacco Control (FCTC) recommends that countries eliminate the sale of kiddie packs and single sticks. In addition, Article 16.3 of the WHO’s Framework Convention on Tobacco Control (FCTC) [19] states that comprehensive policies and effective enforcement strategies are required to stop the sale of single-stick cigarettes and kiddie packs. In 2012, 84 countries (of the FCTC) had policies to prevent the sale of single-stick cigarettes or kiddie packs [20].

The government of South Australia was the first in the world to establish a ban on kiddie packs in 1986 [21], followed by Canada in 1994 [22]. In Asia, Singapore (2002), Brunei (2005), Laos (2009), Malaysia (2010), the European Union Tobacco Products Directive (2014/40/EU) Europe (2014) [10], Cambodia (2015), and Vietnam (2016) had banned kiddie packs from the market as a measure to prevent teens from smoking [23]. The effectiveness of tobacco control measures in many nations has been largely attributable to price-based interventions that lower consumption [10]. Even with the enforcement of the ban, the tobacco industry has tried to reintroduce kiddie packs citing reducing contraband cigarette use as a reason [24]. This contradicts the current tobacco control policies of many countries to prevent minors and teens from accessing and buying cigarettes, as stated previously.

Two reviews were found to publish on the effects of tobacco package quantity on consumption. The first review found articles concerned about the cigarette length [25], and the latter discussed the tobacco companies’ rationale for introducing or changing package amount and packaging type in an effort to influence consumer behaviour; the search was limited to Truth Tobacco Industry Materials (TTIDs) documents [26].

## 2. Evidence Gap

Given the dearth of published information on the direct influence of cigarette pack size is currently uncertain, an evidence-based review of the impact of kiddie cigarette packs on smoking is needed to provide reliable evidence to assist policymakers and stakeholders in formulating new policies and strengthen existing strategies related to the kiddie packs. This would reduce the burden of the government on the health care cost associated with treating diseases caused by tobacco and the human capital lost due to tobacco-attributable mortality and mortality in the future [4]. To our knowledge, no published systematic review addresses our questions, (1) What is the link between kiddie packs on initiation of smoking in the general population? (2) What is the link between kiddie packs on the urge/tendency to buy cigarettes in the general population? (3) What is the link between kiddie packs and an attempt to reduce cigarette consumption among current smokers? (4) What is the prevalence of smoking kiddie packs among current smokers? Therefore, this review was conducted to identify the link between kiddie packs with initiation of smoking, urge/tendency to buy cigarettes in the general population, attempt to reduce cigarette consumption and the prevalence of smoking kiddie packs among current smokers.

## 3. Research Objective

This review aims to identify the link between kiddie packs and smoking. The objectives are:

1. To identify the link between kiddie packs specifically on the initiation of smoking, urge/tendency to buy cigarettes in the general population
2. To identify the link between kiddie packs and attempt to reduce cigarette consumption among current smokers
3. To determine the prevalence of smoking using kiddie packs among current smokers

We conducted a systematic review based on our published protocol [27]. We followed the Preferred Reporting Items for Systematic Reviews and Meta-Analyses (PRISMA) guidelines in conducting and reporting the results of this systematic review (Supplementary Materials Table S1).

## 4. Materials & Methods

### 4.1. Eligibility Criteria

We planned to include all randomized controlled trial, quasi-experimental and experimental studies observational cross-sectional, case-control and cohort studies in this review. We used PECO acronym where P is population specifically any individual with the attempt for initiation of smoking or the urge/tendency to buy kiddie packs in the general population, E is exposure which were those who buy/consume kiddie packs, C is comparators for the impact of kiddie packs was compared with regular sized cigarette packs and O is outcomes which are those mentioned in the objectives above.

### 4.2. Study Design

In this review, we only found three cross-sectional studies for inclusion in this review [18,21,28]. Hence, we made a post-hoc decision to include other types of observational studies, namely qualitative analysis and hypothetical modelling, that addressed the same kind of intervention and outcome. We included original articles in this review without restriction on the publication dates. We included the population-based studies that had cigarettes available for consumption/sale in small cigarette pack sizes compared to standard/larger packs.

### 4.3. Search Strategy

We searched electronic databases to identify all published studies that could be considered eligible for inclusion in this review. We hand searched unpublished studies for inclusion in this review. We used terms such as “cigarette” and “mini pack” OR “kiddie packs” OR “small packs” and “Smoking initiation” OR “impulse to buy” OR “urge to buy” OR “tendency to buy” OR “smoking reduction” as a basis for keyword search. A detailed keyword search is depicted in Table S2 (Supplementary Materials). The literature search identified potential studies in all languages. We searched five databases, including PubMed, EMBASE, The Cochrane Central Register of Controlled Trials (CENTRAL), Web of Science and Scopus, on 27 November 2021. The snowball sampling technique was used to find related studies from the references section of the studies found. The reference from the systematic review or scoping reviews pertaining to the effects of cigarette package size on consumption was also performed. 

We retrieved and reviewed all full texts of potentially relevant articles if the title or first few pages contained information about research on kiddie packs, small cigarette packs, pack size or factors that influence smoking initiation, smoking consumption, or urges/tendencies to buy cigarettes, with regards to kiddie packs.

### 4.4. Study Selection

**Figure 1:**
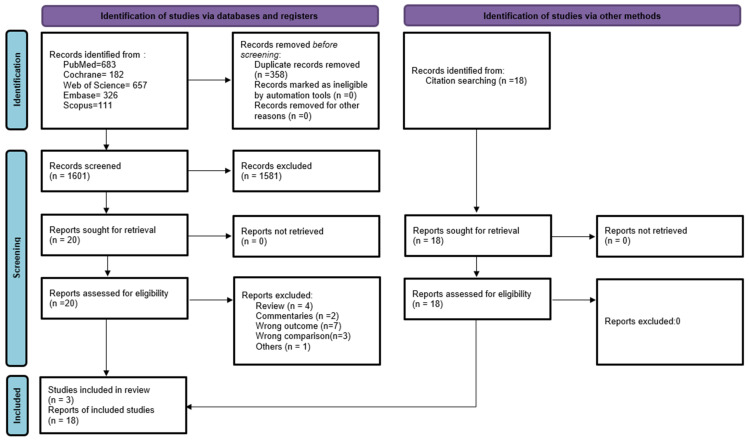
PRISMA 2020 flow diagram. Diagram of searches performed and the number of articles returned and examined at each stage.

Initial search revealed, a total of 1959 potential papers where 1601 articles were obtained after removing duplicates. Four pairs of reviewers (H.M.R. & M.Y.J.L., W.S.R.H. & N.L.A.M., T.A.S. & T.G.R.L., H.A.A.H. & M.R.R.) independently screened the titles and abstracts according to the inclusion and exclusion criteria. Any disagreements were resolved through discussion with the ninth author (N.A.M.). After the titles and abstracts screening, 20 full-text articles were retrieved, and 18 additional articles were identified via cross references. Three pairs of reviewers independently reviewed the full text of these additional articles. Any disagreements were resolved through discussion or referred to the ninth author as a referee (N.A.M.). Of these, twenty-one articles met the inclusion criteria for this review. A detailed description of the process is described in Figure 1.

### 4.5. Data Extraction

Data extraction and analysis were carried out independently by two pairs of reviewers (H.M.R. & M.Y.J.L., T.A. & T.G.R.L.). Another two pairs of review authors independently extracted data using a standardized data extraction form containing information about the authors, year of publication, place of study, study design, number of respondents, type of packaging, outcome and the 16-item quality assessment tool (QATSDD) score (Table 1). 

Two review authors (H.M.R. & M.Y.J.L.) independently screened the studies for inclusion Another two authors (T.A.S. & T.G.R.L.) independently retrieved the full text of the included studies. The authors then undertook a thematic synthesis of the collated results. Themes were prespecified a priori categories from the previous research but were adapted on the basis of the information reported in the included studies [29]. Data were thematically analysed on the link between kiddie packs on initiation of smoking, urge/tendency to buy cigarettes, the prevalence of smoking (using kiddie packs), and attempts to reduce cigarette consumption.

**Table 1:** Summary findings.

Study	Study Type	Sample Size	Intervention (Size of Packaging)	Population	Outcome	QATSDD Score	Sponsorship Status by Tobacco Company
Marti & Sindelar (2015) [30]	Computer-based online survey	N = 868 (survey) N = 593 (focus group)	standard (20 units), smaller (10 units), and larger (30 units) packs	recruited adults from the Yale School of Management eLab (elab.som.edu), USA sample was restricted to those who correctly answered a quality-control question	- *Attempt to reduce cigarette consumption* ∘ About one-third of current smokers would be interested in buying a smaller pack of 10 cigarettes. Most reported consumption regulation as their main reason for choosing a smaller pack. - *Urge/tendency to buy cigarettes (price affordability and convinient to carry)* were the other reason to buy kiddie pack ∘ These smokers are willing to pay a premium for the relatively smaller pack, which is consistent with a demand for a pre-commitment device. ∘ Results from regression models show that preferences for pack sizes match the current consumption of cigarettes. ∘ However, smokers who are interested in quitting and have a higher degree of self-control prefer smaller packs. - No empirical evidence on whether smaller packs are attractive to young adults.	71%	Not sponsored by a tobacco company
Farrell, Fry & Harrus (2011) [31]	Hypothetical modelling count data processes	N = 5766	5, 10.15 and 20 cigarettes per weekdays	Aged 16–74 years living in England	- *Attempt to reduce cigarette consumption* ∘ This article hypothesized the importance of pack sizes on cigarette consumption in a given period based on data from the Health Education Monitoring Survey (HEMS) 1998 ∘ Smokers regulate their consumption according to the size of available packets. ∘ The estimation results suggest that the (expected) number of cigarettes smoked by a typical smoker is 10 per day—equivalent to the amount contained in the smallest packet that consumers can purchase in England. ∘ The results suggested that the government allow cigarettes to be sold in smaller packs to reduce cigarette consumption.	74%	Not sponsored by a tobacco company
Office Tobacco Control (2006) [18]	Cross-sectional study	N = 310	Pack of 10 vs. pack of 20	Aged >8 years living in Ireland	- *Urge/tendency to buy cigarettes (price affordability)* ∘ although, overall, 72% of the smokers bought a pack of 20’s compared to a pack of 10’s, 80% of the smokers age was below 18 years old (age 12–17) preferred to buy a pack of 10’s compared to a pack of 20’s. ∘ These teenage smokers were the price-sensitive group. ∘ Half of the 10’s pack buyers said that they are fairly or very unlikely to continue smoking with a 50% increase in price and 77% of all 10-pack smokers said they are fairly or very unlikely to continue smoking with a 100% increase in price ∘ if the price of the 10’s pack remains the same, 94% of 10’s pack buyers were very or reasonably likely to continue smoking.	52%	Not sponsored by a tobacco company
Wilson et al. (1987) [21]	Cross-sectional study	N = 118 adolescent N = 288 adults	Packets of 15	Adolescents aged 14 and 15, Adelaide, Australia	- *Urge/tendency to buy cigarettes (price affordability and concealment)* were important purchasing factors among adolescents. ∘ Conducted in the Adelaide metropolitan area ∘ 56.3% of adolescent smokers had purchased packets of 15 in the month prior to the survey vs. 8.8% among adult smokers	50%	Not sponsored by a tobacco company
LBC, Levy J & Wood M. (1995) [32]	A qualitative study (In-depth interview)	N = 20	Packet of 10	Adults aged 21–29-year-old, specific brand smokers, New York, NY, USA	- *Urge/tendency to buy cigarettes (price affordability, small size, attractive appearance*) ∘ Several of smokers said they would buy 10’s instead of traditional packs ∘ Purchase interest was driven by: Expected lower price Compactness of the pack Uniqueness of the products	31%	Sponsored by a tobacco company
Phillip Morris & Lopez (1992) [33]	Qualitative study-(one-on-one interviews)	N = 36	Packet of 10’s	Smokers of a specific brand and competitive smokers-women, in Orlando, FL - White and African-American - Ages of 18–40	- *Urge/tendency to buy cigarettes (small size, convenient to carry)* ∘ Women were motivated to purchase a 10 pack as the pack was seen as more discreet, cute and easy to fit into places like purses or pockets.	21%	Sponsored by a tobacco company
Marketing Perception & Wolf (1993) [34]	Qualitative, twelve triads	N = 12 triads, conducted 11–12 August 1993	Special Packet of 10’s	Adults aged 25–44 in Chicago, IL, smoke non-menthol, full flavour or lights, kings or 100’s	- *Urge/tendency to buy cigarettes (price affordability, convenient to carry, small size, attractive appearance)* ∘ Low price-as the way to experiment with a new brand ∘ Stylish look ∘ Easier to carry ∘ More discreet - *Attempt to reduce cigarette consumption*	21%	Sponsored by a tobacco company
Gomez & Guevara (1993) [28]	Cross-sectional study (Consumer Research Report) interview	N = 1000,	14’s pack users	Male and female smokers who claimed to smoke at least 5 cigarettes per day, in Puerto Rico	- Primary reasons for 10’s and 14’s pack size preference were: ∘ *Urge/tendency to buy cigarettes (price affordability)* ▪ Cost (more economical /cheaper) ∘ *Regulate cigarette consumption* ▪ One smokes less with a smaller pack size - Usage of 10 packs declined significantly since 1992 from 11.1 to 7.9%. This happened in the San Juan region and among smokers under 35 years of age - However, the share for the 14’s pack was high (21%)—continues to skew to males and 18–24-year-olds	17%	Sponsored by a tobacco company
Kapuler Marketing Research (1985) [35]	Beta qualitative research	N = 43 uniFocus interviews, Pittsburg, Pennsylvania on 8 and 9 May 1985. N = 10/43 in a group discussion, 9 May 1985	10’s pack and 25’s pack cigarette	Women, aged between 18 to 24, have at least 4 packs of cigarettes per week.	- The women were shown visuals and alternative package configurations and probed for imagery and opinions. - Reasons for preference 10’s pack - compared to 25’s pack were: ∘ *Urge/tendency to buy cigarettes (price affordability, convenient to carry, attractive appearance)* ▪ A stylish look ▪ Ease of carrying ▪ Lower price ▪ Trial/sample pack ∘ *Attempt to reduce cigarette consumption* ▪ Help to cut down on smoking ∘ Disadvantages ∘ A heavy smoker would have to make several trips to the stores - Reasons for 25’s pack ∘ Convenience ∘ Extra cigarettes that could act as cushion and value ∘ Disadvantages: ∘ Bulkiness ∘ Too many cigarettes for a light smoker	26%	sponsored by a tobacco company
Carter SH, 1986 [36]	Qualitative study	N = 8 focus group discussion	12’s pack cigarette	Philadelphia Group 1: females, aged 18–24, N = 10 Group 2: males, aged 18–24, N = 8 Group 3: females, aged 26–35, N = 8 Group 4: males, aged 26–35, N = 7 Columbia Group 1: females, aged 18–24, N = 10 Group 2: males, aged 18–24, N = 10 Group 3: females, aged 31–50, N = 9 Group 4: males, aged 31–50, N = 8	- *Reduce cigarette consumption* - *Urgency/tendency to buy cigarettes (price affordability, convenience to carry, attractive appearance)* ∘ Convenience ∘ Unique ∘ Overall appearance ∘ Less price	36%	Sponsored by a tobacco company
Paul A. Warner Assoc. 1990 [37]	Qualitative study	N = 6 focus group 3 in Chicago 3 in Atlanta	10’s pack	Black adult smokers ages 18 to 24, in Chicago and Atlanta, smoke 5 or more cigarettes per day Not attended college <$20,000 annual household income	- *Urge/tendency to buy cigarettes (price affordability, convenient to carry)* ∘ 10’s pack is the best alternative for occasional smokers ∘ Most likely to be selected by heavy smokers when they are: ▪ low on pocket funds ▪ going out to a club or party where the 10-pack is more convenient to carry in a pocket or small purse	33%	sponsored by a tobacco company
Gomez & Morales (1996) [38]	Qualitative study -Island Tracking Study May 1996	N = 1000, random interviews among smokers	10’s, 14’s and 20’s pack	Male and female smokers, 18 to 65 years old in Puerto Rico, smoke at least 5 cigarettes a da	- Urge/tendency to buy cigarettes (price affordability, convenient to carry) - Attempt to reduce cigarette consumption ∘ Reasons for 10’s and 14’s pack (top 3) ▪ More economical/cheaper ▪ Smoke less ▪ Easier to carry/store	55%	sponsored by a tobacco company
Burke Marketing Research. Package size Evaluation study (1983) [39]	Qualitative study	N = 396	10, 12, 25, and carton of 5 packs	Male and female smokers in a mall in US, 21 years and above	- Favourite package size is 20, an alternative is 25 per package - 25’s is preferable over 10’s or 12’s on a measure of purchase intent and most likely to buy - Reasons for 10’s pack and 12’s pack cigarettes: ∘ *Urge/tendency to buy cigarettes (small size, convenient to carry)* ▪ Convenient package size ▪ Small ▪ Easy to carry ∘ *Attempt to reduce cigarette consumption* ▪ Reduce cigarette consumption ▪ Limit/ cut down on smoking (12%) - 33% of smokers gave positive purchase intent for the 12-count size and 25% for 10 count size	45%	sponsored by a tobacco company
Generation Idea. (1982) [40]	Qualitative study, Semi-rigid package study. A qualitative exploration of consumer reactions to a new type of packaging for cigarettes.	N = 4 focus groups	12’s pack	Panelist of Smokers - Different segments of smokers by the brand of cigarette smoked and by the type of packaging preferred In Atlanta, USA	- *Urge/tendency to buy cigarettes (price affordability, small size, attractive appearance)* ∘ Small size pack is cute but holds too few cigs (easy to lose) ∘ smaller package only appropriate for the social smoker ∘ good for evening use (fit better into the evening bag/provide just enough for smoking after dinner) ∘ Affordable	17%	sponsored by a tobacco company
Paul A. Warner Assoc. (1990) [37]	Qualitative study	In 4 focus group sessions, Adult smokers	10 packs and 20 packs	Smokers in Cleveland, Ohio	- most respondents admit that they have never wished that cigarettes were available in package sizes other than the standard 20’s, and prefer a larger pack - 10 pack reaction: - *Attempt to reduce cigarette consumption* ∘ this size would be helpful to people who are trying to cut down or quit smoking - *Urge/tendency to buy cigarettes (convenient to carry, small size)* ∘ easy to carry ∘ cannot share with others-easier to remove the cigarettes ∘ easy to fit into a purse/pocket	26%	sponsored by a tobacco company
Market Research Document, (1991) [41]	Segmentation—Phase I—Focus Group Research—Ontario/Quebec	N = 116	with 15-, 20-, and 25-packs.	Canadian smokers, Canada	Reason for 15’s pack: - *Urge/tendency to buy cigarettes (price afoordability)* ∘ beneficial to those with the least money (youth, beginner smokers, the poor) and was frequently referred to as the ‘poverty pack’, with people admitting to having purchased 15’s with a self-conscious and self-deprecating laugh. - *Attempt to reduce cigarette consumption* ∘ associated with those attempting to quit smoking through gradually cutting down. - for those who may desire an occasional change in brand.	24%	Sponsored by a tobacco company
Shoi Balaban Dickinson Research Inc. (1983) [42]	Exploratory research, qualitative study	N = 56 respondents (24 women and 32 men)	package of 10, 15 and 25 cigarettes cigarettes	- all respondents to be in the 18–54-year age range - all respondents to smoke at least one-half pack of filter cigarettes per day	Response for 10 cigarettes: for special occasional use - *Urge/tendency to buy cigarettes (price affordability)* ∘ most respondents said that the 10-pack appeared to be more bargain than the current pack. ∘ it is the potential to be viewed as a trial size that one would purchase to try a new brand. Response for 15 cigarettes: - *Attempt to reduce cigarette consumption (to cut down on smoking)* - consider 15 cigarettes as too small for those who smoke more than 15 cigarettes per day	29%	sponsored by a tobacco company
Cox AR. (1983) [43]	Qualitative study	N/A Detroit and Tanpa - at the retail outlet only.	12 packs	In the USA	- 12’s pack did not generate much purchase. - Useful for temporary promotional purposes to generate occasional trials. - smokers who are interested in cutting down/occasional light smokers, smokers who have not been smoking for a great deal of time would be interested in 12 packs - 79% of the smokers probably and would not probably buy 12 pack - Over half of the buyers said they would probably or definitely repurchase a 12-pack. - Skewed towards young adults(mean) and lower-income smokers. - Some of the reasons for buying 12 pack are: *Urge/tendency to buy cigarettes (small size, price affordability)* ∘ Cute/small ∘ Not enough money/less expensive ∘ New/different ∘ Usual brand not available *Attempt to reduce cigarette consumption* ∘ Easier to cut down the smoking volume (smoking cessation) Conclusion: Twelve packs do not represent a significant volume opportunity	14%	sponsored by a tobacco company
Ellison Quarterly Research (1991) [44]	Qualitative study, 23 and 24 October 1991, Boston Metro Area (Framingham, Massachusetts)	N = ? 6 groups	10 or 14 cigarette pack	3 groups- grouped male non-methol (a specific brand) smokers, age range 18–34 yo 2 groups-female non-methol (a specific brand) smokers, age range 18–24 yo 1 group-male smokers of competitors to RJ brands	- Approximately half (or even 60–65%) able to buy a half-pack cigarette - Smokers prefer 10’s because [*urge/tendency to buy cigarettes (price affordability, convenient to carry)*] ∘ Less expensive ∘ Ease of carrying	26%	Sponsored by a tobacco company
Phillip Morris & Stern D (1990) [45]	Qualitative study	N = 763 adult smoker N = 50 (a specific brand) purchaser	10’s pack	Adult smokers, In LA County, California	- 10’s purchasers tend to buy because they like the pack. [*Urge/tendency to buy cigarettes (attractive appearance, small size, convenient to carry, price affordability)*] ∘ Convenient size ∘ Small ∘ Just wanted to try ∘ Less expensive - 38% of a pack of 10’s-purchasers would stop buying if ten packs were no longer sold.	33.3%	sponsored by a tobacco company
Causey RA (1982) [46]	Qualitative study	N= 8 groups (8–10 per group)	10’s pack	Smokers (current smokers and ex-smokers)	- 10’s packaging is well-liked - Reasons for 10’s pack ∘ *Urge/tendency to buy cigarettes (price affordability, convenient to carry, concealment)* ▪ Less expensive ▪ Easy to carry around ▪ Not supposed to be smoking (reason for youngsters)-easier to hide (youngsters) ∘ *Attempt to reduce cigarette consumption* ▪ Smoke less	33.3%	sponsored by a tobacco company

### 4.6. Assessing the Study Methodological Quality and Bias

The included studies are diversed in terms of study designs, data collection methods, data types, and analytical methods used. Due to this, we decided to use QATSDD compared to ROBINS-I and Rob2 as stated as options for risk of bias of assessment in our protocol. It is a validated tool developed by Sirriyeh et al. [47], and it was used by other studies such as Vyth et al. [48], Hughes et al. [49] and Adam & Jensen [50].

The tool consists of 16 criteria (14 of these criteria apply to qualitative studies, 14 apply to quantitative studies, and all 16 apply to any mixed methods papers), each with a score ranging between 0 and 3, with three being the best. The maximum score for mixed papers is 48 and 42 for qualitative or quantitative. Aspects of clarity were reflected in the description of aims and setting, data quality, method of analysis and self-evaluation. Two independent pairs of reviewers assessed the fulfillment of 16 criteria for each publication based on the information provided in the full assessed texts and a score corresponding to the level of satisfactory attainment of the criteria outlined by QATSDD tools.

The scores were added and divided for each full text by the maximum possible score to report the paper’s overall quality score. A score of 0 was given if the authors had not included the level of detail required to make a judgement for quality criteria.

The first pair of reviewers assessed all studies, and inter-rater reliability was determined by rating a random selection of the second pair of reviewers and the third pair of reviewers. Disagreements were resolved through discussion and abstract screening. Quality of papers was used to guide the review in terms of implications of each study and to inform the conclusion made from outcomes. Study quality was categorised based on the GRADE approach [51]. Scores of 0–25% were considered very low quality; 26–50% low quality; 51–75% moderate and scores of 76–100% were considered of high quality [51]. A direct comparison of study quality could be done based on this category and a consistent interpretation of QATSDD could be promaded. Lower-quality studies are more likely to have systematic errors or higher levels of bias [51].

## 5. Results

A formal meta-analysis was not possible due to the heterogeneous nature of study settings, designs and outcome measures. Therefore, studies with a similarly themed component were grouped for narrative synthesis. Data were grouped based on the link between kiddie packs with initiation of smoking, urge/tendency to buy cigarettes, and attempt to reduce cigarette consumption among the population.

### 5.1. Summary of the Main Results of the Included Studies

While searching relevant papers for inclusion in the review, a total of 1601 potential papers were identified after duplications were removed. After the titles and abstracts screening, 38 papers were selected for further screening. Of those, we included 21 studies. Of the 21 studies, 4 studies were conducted independently whereas the remaining 17 studies were conducted by the tobacco companies.

Summary of findings containing information on study type, data collection and analysis and methodological quality were presented in Table 1. 

### 5.2. Methodological Quality and Bias of Included Studies

Scoring for study quality was based on the QATSDD’s 16 criteria and a summary of each study has been included below.

The methodological quality scores of all the included studies range between 14% to 74%, yielding an average quality score of 34.8%. The methodological quality of four studies which were conducted independently [18,21,30,31] ranged from 50% to 74%, with one [21] being low quality and others being moderate in quality [18,30,31]. Meanwhile, the articles conducted by the tobacco industry company ranged from 14% to 55%. Only 1 article was moderate in quality [30] and others were either of poor or very poor quality.

Based on the GRADE approach category, 4 studies were categorised under moderate studies [18,30,31,38] and the majority (11 studies) were under low-quality studies. The quality assessment revealed that research on the topic largely failed to report the rationale for assessment of the reliability of the analytic process (Criteria 14), evidence of user’s involvement in design (Criteria 15), detailed recruitment data (criteria 8), good justification for the analytical method selected (Criteria 13), fit between stated research question and method of data collection (Criteria 11), fit between research question and method of analysis (Criteria 12), representative sample of a target group of a reasonable size (Criteria 5) and Statistical assessment of reliability and validity of measurement tools (Criteria 9) to allow for research to be replicated. Of those studies with scores higher than 50%, 3 [21,30,31] were independent of the tobacco company and 1 was sponsored by the tobacco company [38]. Criteria for which most studies scored low include assessment of the reliability of the analytic process (0.0%), evidence of user’s involvement in design (1.6%), and recruitment data (12.7%). These scores were low due to the inadequate level of detail included by the authors for the paper included.

Table 2 shows the mean and standard deviation of the 16 methodological assessment criteria. Almost all evaluated studies scored an average mean of 2.81 for a “clear description of research setting”. The lowest average scores were found for “assessment of the reliability of analytic process” and “evidence of user involvement in design”; the average scores were 0.00 and 0.05 respectively. The details of the critical appraisal of the included studies are shown in Table S3 (Supplementary Materials).

### 5.3. Study Design and Setting

The studies were different in terms of study type, sample size and target population. Studies were conducted in the United States of America (USA), Australia, United Kingdom (UK), Ireland Canada, Puerto Rico and Paraguay. Sixteen were qualitative studies [32,33,34,35,36,37,38,39,40,41,42,43,44,45,46], three were cross-sectional studies [18,21,28], one was computer based online survey [30], and one was modelling count data process study [31]. The qualitative studies generally did not provide significant tests or impact sizes. Fourteen studies used sample packs [21,28,32,33,36,37,38,39,40,41,43,44,45,46] but the other six used product images [30,31,34,35,37,42]. Only 6 studies detected the link between the kiddie packs and smoking after their introduction into the market [18,21,28,38,45,46]. From these 6 studies, most concluded that the urge to buy cigarettes is the effect of the kiddie cigarette pack in the market. Of those, only 2 studies investigated the link between kiddie packs on teenagers [18,21]. The study reported that more than half the adolescent smokers (age 14 and 15) (56.3%) had purchased a packet of 15 cigarettes in the month prior to the survey compared to adult smokers (8.8%) [21], which revealed that kiddie packs attracted more teenage smoker consumers compared to adults. The study also found that the majority of the consumer gave the price as the reason to purchase a packet of 15’s [21]. The other fifteen studies were designed to detect the potential impact of kiddie packs on smoking.

### 5.4. Participants

The included studies with complete age and sample size information revealed that the participants’ age range from 12 to 54 years old, whereas the sample size ranged from 6 to 5766 participants.

Of the 4 independent articles of the tobacco company, 3 articles conducted studies among adolescents [21,31] and children [18] and others were conducted among adults [30,31]. For tobacco industry company-sponsored study, most of the studies that had complete age information were conducted among adults.

### 5.5. Issues of Quality and Bias in the Included Studies

Wilson et al. [21] used a convenience sample from high schools in Adelaide, Australia. This study focused on adolescents aged 14 and 15 only and limited its survey to only two brands of cigarettes presenting a potential selection bias of the sample; a wider range of age and brands may have provided further insight. The authors justified their choice of study designs with clear methods but there was no discussion around potential confounders for their results.

A study in Ireland used stratified random sampling from 8+ years of living in the Republic of Ireland. The survey did not limit its survey to a specific brand. The authors justified their choice of study design with a clear method but no discussion around potential confounders for their results [18].

Marti & Sindelar (2015) [30] used a computer-based online survey, which involved the recruitment of adults who were smokers and registered to the Yale School of Management Lab, which acted as a platform for online surveys and experiments. This led to a potential selection bias in the sample. They provided the justification for the study design and clear methods and there was discussion around potential confounders for their results.

Farrell, Fry & Harrus (2010) [31] used secondary data from an individually based nationally representative survey involving adults between the ages of 16 to 74 years old living in England. They provided relevant details and used an appropriate approach according to the objectives of their study. Potential confounders from the results were extensively discussed.

Other seventeen studies were conducted by tobacco companies [28,32,33,34,35,36,37,37,38,39,40,41,42,43,44,45,46,52]. Sixteen studies were qualitative studies [32,33,34,35,36,37,37,38,39,40,41,42,43,44,45,46]. All of the qualitative studies limited their survey to limited brands of cigarettes; a wider range of brands may have provided further insights. Also, the target groups were specific to their own objectives which may not have reflected the actual scenario in the general population. Little discussion was provided around potential bias from participants and how it was managed while conducting the focus groups.

Gomez & Moralez (1996) [38] used cross-sectional studies which screened 5976 respondents, out of which 1000 were smokers and interviewed. This study did not limit its survey to certain brands, where it focused on wider brands of cigarettes. The authors provided no other detailed sampling methods, and the study design was not explained. Potential confounders were also not discussed leading to potential bias.

Gomez & Guevara (1993) [28] interviewed respondents in Puerto Rico on smoker’s consumer behaviours. Multiple cigarette brands were included in the study for the diversity of the consumers, but no other detailed sampling methods or study designs were explained. Potential confounders were also not discussed leading to potential bias.

Burke Marketing Research in its Package Size Evaluation Study (1983) [39] explored the possibilities of marketing cigarettes in a new package size. The study design and sample size was poorly described. Convenient sampling was applied within selected shopping malls during the malls’ operating hours. Respondents’ selection criteria were not mentioned.

### 5.6. Outcome of the Included Studies

#### 5.6.1. Urge/Tendency to Buy Cigarettes

Kiddie packs increased the urge to buy cigarettes among smokers which were consistent with several studies discussed below. There were several reasons that influenced the urge to buy cigarettes in kiddie packs.

##### Price Affordability

There were seventeen studies that noted that kiddie packs were cheaper, and this stimulated purchase among smokers [16,18,21,28,32,34,35,36,37,38,40,41,42,43,44,45,46]. Studies [18,32,34], revealed that purchase interest in kiddie packs was driven by expected lower prices. 

##### Convenient to Carry

Another eleven studies revealed that the urge to buy kiddie packs was due to the convenience of carrying them around [30,33,34,35,36,37,38,39,44,46]. A focused group study done by a research company named Paul A. Warner Association [37] showed that kiddie packs are more convenient to carry in a pocket or small purse when going to a club/party. A small qualitative study conducted by another research company which was funded by a tobacco company noted that kiddie packs were good for evening use as they fitted better into an evening bag and the quantity was sufficient for smoking after dinner [39].

##### Small Size

Nine studies revealed the reason for buying kiddie packs was due to their small size [32,33,34,37,39,40,43,44,45]. Women smokers were motivated to purchase kiddie packs as it was seen as more discreet and cuter [33]. Another reason was that the smokers just wanted to try a new product that had a lesser number of cigarettes. Studies [43,45] found that kiddie packs were seen as something new that influenced people to try.

##### Attractive Appearance

Six other studies also found that the urge to buy kiddie packs increased because they were more attractive and stylish [32,34,35,36,40,45].

##### Concealment

Two studies found that the reason for buying kiddie packs was because it was easy to hide. Causey in a qualitative study in Paraguay found that the reason younger smokers bought kiddie packs was due to its ease in being concealed as they were not supposed to be smoking [46]. This was similar to a finding by Wilson et al. [21] which noted that concealment was among the reasons adolescents aged 14 to 15 years old in Adelaide bought kiddie packs.

#### 5.6.2. Attempt to Reduce Cigarette Consumption

We found mixed findings on this outcome. Attempt to reduce cigarette consumption/smoking cessation were among the main reasons for buying kiddie packs. Some of the current smokers preferred kiddie packs as a tool to quit smoking and reduce or maintain a desired consumption level. There were ten twelve studies that discussed attempts to re-duce cigarette consumption [28,30,34,35,36,37,38,39,41,42,46].

Marti & Sindelar (2015) [30] noted that smokers who preferred smaller cigarette packs were more likely to want to quit smoking and more than 70% of smokers purchased kiddie packs to limit their cigarette consumption. Farrel, Fry & Harrus (2011) [31] hypothe-sized that smokers regulated their consumption according to the size of packs that were available. That study also stated that the average number of cigarettes smoked by a typical smoker was 10 sticks per day, which was equivalent to the number of sticks in the smallest packs that consumers can purchase in England. The other nine studies also showed that one of the reasons for kiddie pack preference was to smoke lesser with a smaller pack size. However, there was a study in Ireland that found that consumers prefer to continue buying kiddie packs if there was no increase in price and the majority had no intention to quit smoking [18].

#### 5.6.3. Prevalence of Smoking (Using Kiddie Packs)

We found a study which that reported that more than half the adolescent smokers (age 14 and 15) (56.3%) had purchased a packet of 15 cigarettes in the month prior to the survey, which was higher compared to adult smokers (8.8%) [21]. Another study in Ire-land also revealed that the majority (80%) of adolescent smokers aged 12 to 17 more often purchased kiddie packs (pack of 10 vs. 20 packs) compared to adults (24%) [18]. Both findings revealed that kiddie packs attracted more teenage smoker consumers compare to adults. 

#### 5.6.4. Initiation of Smoking among Population

We did not find any study that discussed the link between kiddie packs and smoking initiation.

## 6. Discussion

We utilised a methodological assessment instrument to evaluate both quantitative and qualitative studies on the relationship between child cigarette packaging and smoking in an objective manner. It is likely that the ratings will be underestimated when research meet the criteria listed in the evaluation tool without clear reporting in the publication. The evaluation revealed that the majority of the research were of poor quality.

This research identified just a small number of studies on the actual link between kiddie packs and smoking. Based on our review, it answers our first objective of identifying the link between kiddie packs and the urge/tendency to buy cigarettes. The majority of included studies found that kiddie packs were purchased because to their lower price, which increased the purchase of cigarettes among smokers. The cheaper prices of cigarette packs encouraged smokers, particularly youths and those with limited incomes, to purchase smaller packs [18,21]. This is consistent with research indicating that lower prices can encourage/attract low-income consumers, primarily adolescents and minors, to purchase kiddie/smaller cigarette packs due to their affordability [53], as it has also been demonstrated that tobacco consumption increased when prices decreased [11,12]. In addition, this study corroborated the present tobacco policies of many nations, which restrict minimum cigarette packet numbers to enhance barriers to use by youngsters [26]. This indirectly discourages adolescent smoking, as smoking has numerous negative impacts on the body. There were two (independently non-funded by the tobacco industry) post-marketing cross-sectional studies focused on the link between kiddie packs and smoking [18,21]. Based on our study, 2 studies demonstrated that young smokers under the age of 18 had a greater propensity to purchase kiddie packs than adults. Wilson et al. [21] conducted a study in the metropolitan area of Adelaide to explore current smoking behaviour (in terms of the desire to purchase) and evaluate new cigarette marketing strategies among 14- to 15-year-old adolescents, in which two prominent brands were supplied in 15-count packets. Compared to adult smokers (8.2%), more than half of adolescent smokers (ages 14 and 15) had purchased a pack of 15 cigarettes in the month before to the poll, according to their research. The primary reason was its reasonable price. Another study indicated that the majority of adolescent smokers between the ages of 12 and 17 (80%) were more likely to purchase kiddie packs (10 packs vs. 20 packs) than adults (24%). The majority of adult smokers began smoking as adolescents [17]. %According to a 2016 survey of 143 adolescent smokers, 70% began smoking between the ages of 12 and 15. Nearly 60% did not even enjoy smoking and unsuccessfully attempted to quit. Another survey of people aged 8 and older in Ireland found that 78% of smokers began before the age of 18 and 53% before the age of 15. This demonstrated that smoking initiation mostly affects children and adolescents [18]. This is particularly concerning because smokers exposed themselves to the negative effects of cigarettes at a very young age, which led to smoking-related ailments [17] and other high-risk behaviours [54]. The economic cost of tobacco is substantial and includes astronomical health care expenses for treating diseases caused by tobacco use. In addition, human capital is lost as a result of tobacco-related disease and mortality [4].

In addition to kiddie packs’ affordable pricing, the desire to purchase them was fueled by their portability and concealability, particularly among women and younger smokers due to their diminutive size [21,33,46,52]. As stated in the preceding paragraph, since the majority of kiddie pack consumers were adolescents, the concealment of kiddie packs from their parents would allow the adolescents to continue smoking as their parents would be unaware of their smoking status. Multiple studies have demonstrated that parental participation is an important protective factor against daily smoking among teenagers [54]. In addition to the engagement of other parties, such as school-based intervention [55], high parental involvement significantly reduced the likelihood of adolescents becoming current smokers [54]. In addition to the tobacco-related disorders that might result from smoking, high-risk behaviour can be a consequence of tobacco use [4]. Studies have found a substantial correlation between alcohol consumption and cigarette smoking [54]. Multiple theories led to the link between alcohol consumption and smoking [54]. The interaction between the role of peer influence, subculture theory, psychological discomfort, and sociocultural influence may account for the concurrent use of cigarettes and alcohol. In addition, studies have demonstrated a correlation between smoking and school absenteeism [54]. Several research have shown contradictory results about this association. Absenteeism may be caused by smoking-related sickness or delinquent behaviour that leads to smoking. In addition, research indicates that smoking is connected with bullying [54]. On the basis of these arguments, it is vital to discourage adolescents from smoking at a young age, as smoking is associated with numerous negative health effects and high-risk behaviour. Current tobacco policies deter adolescents from smoking by limiting their ability to obtain cigarettes and imposing a high cost on tobacco products. The desire to purchase kiddie packs was also influenced by their appealing appearance. Tobacco control efforts in a number of nations include the sale of plain packaging to deter consumers from being attracted to the appearance of cigarette packs. As resources are extremely limited, however, additional high-quality research examining the influence of kiddie packs on the desire to buy cigarettes are required.

This evaluation also uncovered contradictory evidence for the second objective: link between kiddie packs and attempts to reduce cigarette smoking among current smokers, although additional high-quality research is required to verify this. Literature indicates a correlation between cigarette pack size and the number of cigarettes smoked [10]. Daily self-reported smoking was positively correlated with pack size [10]. Certain smokers utilise small packs as a way for regulating their cigarette consumption [10]. Our analysis revealed that one of the motivations for purchasing kiddie packs was to limit cigarette usage. Based on the regression models, Marti and Sindelar (2015) [41] found that pack size preferences corresponded to current cigarette use, so smokers who wished to quit and those with a stronger degree of self-control tended to purchase small packs. Wilson et al. (1987) [39] found that 10.5% of adolescent smokers purchased child packs in an effort to quit. Attempts to reduce cigarette consumption was also cited in the remaining eight qualitative investigations as the reason why smokers purchase kiddie packs. Despite studies indicating that kiddie packs cut cigarette consumption among current smokers, the quality of these studies is relatively low. Prior to reaching a conclusion, additional high-quality investigations are required. It is standard practise in the food industry to minimise the size of containers so as not to increase the price of the products consumers purchase, and this practise is effective. However, there is a counterargument stating that tobacco companies may limit the amount of smokes each package in order to conceal the price increase. Tobacco companies asserted that their marketing efforts had no effect on the initiation of tobacco use among young people and do not affect the general demand for tobacco products [56]. According to several studies, the marketing operations of the tobacco business have been the primary factor in attracting young people to tobacco use, deterring users from quitting, and increasing consumption among users [56]. Attempts to reduce or raise the amount of cigarettes per pack are often marketing strategies employed by one brand in competition with another to sell more cigarettes and boost profits. Wilson et al. [21] discovered that 15-count packages are marketed primarily to teens. Research done in Ireland indicated that 94% of kiddie pack purchasers claimed they were very or somewhat likely to continue smoking if cigarette prices remained the same [18]. If the price keeps the same, the kiddie pack had no effect on smoking rates. According to this study, pricing is the most effective method for regulating tobacco usage and addiction, particularly among price-sensitive young people. A price rise discourages youth from experimenting with tobacco products and discourages many current smokers from continuing. 77% of purchasers of a 10-pack stated that if the price of cigarettes increased by 100%, they would be unlikely to continue smoking. The introduction of a pack of 10 was aimed towards particularly sensitive adolescents. Thus, the present tobacco control regulations in many nations are supported for stopping this price-sensitive demographic from purchasing cigarettes. For instance, according to Malaysia’s 2012 International Tobacco Control Report, tobacco taxes and prices have not risen at a rate sufficient to counterbalance income growth [57]. Therefore, the most effective method for reducing cigarette consumption is to drastically increase cigarette taxes. This will not only reduce the number of adult smokers, but it can also dissuade young people from starting to smoke. A tax increase will result in a 4% decrease in tobacco usage in high-income nations and a 5% decrease in low- and middle-income countries [4]. Access to kiddie packs is impeded by high taxes; enhancing the current tobacco policy control will continue to aid in preventing adolescents from smoking.

The included studies did not evaluate the impact of kiddie packs on the prevalence of smoking, nor did they discuss the introduction of kiddie packs to stimulate smoking initiation among non-smokers or among juveniles. However, there is evidence that teen smokers purchase kiddie packs far more frequently than adult smokers [18,21].

Even though the majority of research were of poor quality, the findings could be useful for policy implications. Compared to kiddie packs, which are cheaper and smaller, packs of 20 cigarettes with a higher price are a more effective strategy to fight the current problem [58]. This action will not solve the illegal cigarette trade, which we believe should be addressed in other ways, such as by improving enforcement and imposing harsher penalties on the traders [17,23].

In accordance with the Framework Convention on Tobacco Control (FCTC) of the World Health Organization (WHO) [19], many nations have outlawed the sale of single sticks and kiddie packs. On the other hand, numerous initiatives to reintroduce kiddie packs in an effort to prevent illegal cigarette sales are controversial. It was debated if it is preferable for smokers to purchase juvenile packs at a lower price than to purchase illegal cigarettes. However, our research indicates that kiddie packs tend to boost the desire to purchase cigarettes, particularly among adolescents.

The average quality score for all papers in this study was 34.8%, indicating that these investigations were of poor quality. Therefore, additional high-quality research is required for a definitive conclusion about the link with kiddie packs and smoking.

In general, kiddie pack research is often limited. We are unsure of the link between kiddie packs and smoking initiation. The majority of the documents were dated in the 1980s and 1990s, which may not reflect the current state of affairs. These publications were also sponsored by the industry, which can introduce bias into the aims and desired outcomes. Due to these circumstances, we were unable to evaluate the accuracy of certain research findings. 

## 7. Conclusions

Based on the available evidence in this systematic review, there is evidence that kiddie packs are very preferentially bought by teenage smokers compared to adult smokers. In addition, there is some evidence that kiddie packs increase the urge or tendency to buy cigarettes, especially among both smoking youth and adults. The evidence supported the current tobacco policies of many countries in preventing minors and teenagers from purchasing cigarettes. We also found mixed evidence on whether kiddie packs reduce cigarette consumption. However, this study did not find conclusive evidence of the impact of kiddie packs on the initiation of smoking.

With the paucity of evidence-based research used in published studies and the absence of any clinical trials; this review highlights the need for better quality research data in the future. More independent tobacco company-sponsored studies are needed to identify the direct influence of kiddie cigarette packs on smoking so that it could help policymakers make a decision on creating or strengthening the existing tobacco control policies to make sure teenagers and minors are protected from the harmful effects of cigarettes. 

## 8. Limitations

These findings should be interpreted with caution: (1) The methodological quality of some studies raises questions as most were low in quality due to insufficient information. (2) the link between kiddie packs and smoking is not always the focus of the included studies. (3) causality findings can be deemed to be hasty as they are based on cross-sectional and qualitative study design.

## 9. Implication for Practice and Implication for Research

This review uncovered various concerns. First, most of the included studies were of poor quality and were not done by independent researchers (those who were not financed by a cigarette corporation), resulting in a conflict of interest. It is crucial to conduct independent research to draw more accurate conclusions about the outcome. In addition, further high-quality studies are required to assess the link between kiddie packs and smoking clearly and to replicate the method of doing the results to produce more useful data. Moreover, the studies revealed limited outcomes. There is evidence of an urge to buy kiddie packs, particularly among adolescents under 18 years of age due to their affordability. Thus, the current tobacco control measures, which prohibit the sale of kiddie packs, prevent price-sensitive youths from beginning smoking at a very young age. This would lower the burden on the government, as the economic consequences of tobacco use are large and include high health care expenses for treating diseases induced by tobacco use as well as lost human capital due to tobacco-attributable morbidity and mortality [4].

## Figures and Tables

**Table 2:** List of criteria used to assess the methodological quality of the studies included in the review.

#	Criteria	Mean	S.D
1	Explicit theoretical framework	1.38	0.97
2	Statement of aims/objectives in the main body of the report	2.38	0.74
3	A clear description of the research setting	2.81	0.51
4	Evidence of sample size considered in terms of analysis	1.10	1.22
5	A representative sample of the target group of a reasonable size	0.81	1.08
6	Description of the procedure for data collection	1.62	0.74
7	The rationale for the choice of data collection tool(s)	0.81	0.75
8	Detailed recruitment data	0.38	0.86
9	Statistical assessment of reliability and validity of measurement tool(s) (Quantitative only)	0.86	1.21
10	Fit between stated research question and method of data collection (Quantitative only)	2.00	1.29
11	Fit between stated research question and format and content of data collection tool, e.g., interview schedule (Qualitative only)	0.86	0.95
12	Fit between research question and method of analysis	0.67	0.97
13	Good justification for the analytic method selected	0.57	0.87
14	Assessment of reliability of analytic process (Qualitative only)	0.00	0.00
15	Evidence of user involvement in the design	0.05	0.22
16	Strengths and limitations critically discussed	0.48	0.68

## Data Availability

Not applicable.

## Supplementary Materials

The following supporting information can be downloaded at: https://www.mdpi.com/article/10.3390/ijerph191912051/s1, Table S1: PRISMA 2020 Checklist (from [59]); Table S2: Search terms used; Table S3: Results of the included studies based on the QATSDD.

## Abbreviations

PRISMA: Preferred reporting items for systematic reviews and meta-analyses; QATSDD: A 16-item quality assessment tool; SD: standard deviation.
