# Supplementary material for: Small/Kiddie Cigarette Packaging Size and Its Impact on Smoking: A Systematic Review
Source: Int J Environ Res Public Health. 2022 Sep 23;19(19):12051. doi: 10.3390/ijerph191912051 (PMC9566128; doi:10.3390/ijerph191912051)
Supplement: Supplementary file 1 [file ijerph-19-12051-s001.zip › Table S2 Search terms used.pdf]

Table S2. Search terms used. Contains search terms used for database literature search.

Key Search Strategy	Search Date
**The databases include Pubmed, Scopus, Web of Science, The Cochrane Central Register of Controlled Trials(CENTRAL) and EMBASE.**<br><br>**Keywords for Pubmed:**<br>((smoke* OR tobacco* OR cigarette* OR smoking OR (smoking[MeSH Terms]) OR (cigarette smoking[MeSH Terms])) AND (("small pack*" OR "kiddie pack*" OR "mini pack" OR "small box*" OR "12-pack" OR "12's" OR "14-pack" OR "14's" OR "10-pack" OR "10's" OR "5-pack" OR "5's" OR ″15-pack″ OR ″15′s″ OR "pack sizes" OR "pack size" OR "package size")) AND ("smoking initiat*" OR "urge to buy" OR "impulse" OR "smoking reduc*" OR "intend" OR "desire" OR "consump* OR (smoking reduction[MeSH Terms]) OR (smoking behaviors[MeSH Terms]) OR (smoking habit[MeSH Terms]))<br>**Keywords for Cochrane:**<br>(("small pack*" OR "kiddie pack*" OR "mini pack" OR "small box*" OR "15-pack" OR "15's" OR "12-pack" OR "12's" OR "14-pack" OR "14's" OR "10-pack" OR "10's" OR "5-pack" OR "5's" OR "pack sizes" OR "pack size" OR "package size" OR packet)) AND<br>(smoke* OR tobacco* OR cigarette* OR smoking) AND<br>("smoking initiat*" OR "urge to buy" OR "impulse" OR "smoking reduc*" OR "intend" OR "desire" OR "consump*")<br>**Keywords for Embase:**<br>('small pack*' OR 'kiddie pack*' OR 'mini pack' OR 'small box*' OR '15-pack' OR '15` s' OR '12-pack' OR '12` s' OR '14-pack' OR '14` s' OR '10-pack' OR '10` s' OR '5-pack' OR '5` s' OR 'pack sizes' OR 'pack size' OR 'package size' OR packet) AND<br>('smoking initiat*' OR 'urge to buy' OR 'impulse' OR 'smoking reduc*' OR 'intend' OR 'desire' OR 'consump*) AND (smoke* OR tobacco* OR cigarette* OR smoking OR 'smoking'/exp OR 'cigarette smoking'/exp)<br>**Keywords for Web of Science:**<br>((ALL=((("small pack*" OR "kiddie pack*" OR "mini pack" OR "small box*" OR "15-pack" OR "15's" OR "12-pack" OR "12's" OR "14-pack" OR "14's" OR "10-pack" OR "10's" OR "5-pack" OR "5's" OR "pack sizes" OR "pack size" OR "package size" OR packet)))) AND ALL=((smoke* OR tobacco* OR cigarette* OR smoking))) AND ALL=(("smoking initiat*" OR "urge to buy" OR "impulse" OR "smoking reduc*" OR "intend" OR "desire" OR "consump*"))<br><br>**Keywords for Scopus:**<br>(TITLE-ABS-KEY ({small pack}<br>OR {kiddie pack*} OR {mini pack} OR {small box*} OR {15-pack} OR {15's} OR {12-pack} OR {12's} OR {14-pack} OR {14's} OR {10-pack} OR {10's} OR {5-pack} OR {5's} OR {pack sizes} OR {pack size} OR {package size} OR packet) AND TITLE-ABS-KEY (smoke* OR tobacco* OR cigarette* OR smoking) AND TITLE-ABS-KEY ("smoking initiat*" OR "urge to buy" OR "impulse" OR "smoking reduc*" OR "intend" OR "desire" OR "consump*"))	27 November 2021
