# Supplementary material for: Small/Kiddie Cigarette Packaging Size and Its Impact on Smoking: A Systematic Review
Source: Int J Environ Res Public Health. 2022 Sep 23;19(19):12051. doi: 10.3390/ijerph191912051 (PMC9566128; doi:10.3390/ijerph191912051)
Supplement: Supplementary file 1 [file ijerph-19-12051-s001.zip › Table S3 QATSDD summary .pdf]

Table S3. Results of the included studies based on the QATSDD.

Study	Theory	Aims/ Objectives	Setting	Sample Size	Sample Represent	DC Procedures	DC Tools	Recruitment Data	Assess Rel & Validity (Quant Only)	Fit: RQ and DC (Quant Only)	Fit: RQ ad DC (Qual Only)	Fit: RQ and Analysis	Justify Analysis	Reliability Analysis (Qual Only)	User Involvement	Strengths and Limitations	Total and % of Highest Possible Score
Burke Marketing Research (1983) [39]	2	3	3	1	1	2	1	2	0	1	-	1	1	-	0	0	**19/42 = 45%**
Carter (1986) [36]	1	3	3	3	1	2	1	0	-	-	1	0	0	0	0	0	**15/42 = 36%**
Causey (1982) [46]	2	2	2	2	1	2	1	0	-	-	0	1	1	0	0	0	**14/42 = 33.3%**
Curtis (1985) [35]	0	3	3	0	0	3	1	0	-	-	1	0	0	0	0	0	**11/42 = 26%**
Cox (1983) [43]	1	1	1	0	0	1	0	0	-	-	0	1	0	0	0	1	**6/42 = 14%**
Ellisom Quarterly Research (1991) [44]	1	1	3	1	1	1	0	0	-	-	1	1	1	0	0	0	**11/42 = 26%**
Farrel, Fry & Harrus (2011) [31]	3	3	3	2	3	3	3	2	3	1	-	2	2	-	0	1	**31/42 = 74%**
Generation Idea (1986) [40]	1	1	3	0	0	2	0	0	-	-	0	0	0	0	0	0	**7/42 = 17%**
Gomez& Guara (1993) [28]	0	2	3	0	0	1	1	0	0	0	-	0	0	-	0	0	**7/42 = 17%**
Gomez& Morales (1996) [38]	2	3	3	3	2	2	1	1	1	3	-	0	1	-	1	0	**23/42 = 55%**
Levy & Wood (1995) [32]	1	2	3	0	0	2	0	0	-	-	2	1	0	0	0	2	**13/42 = 31%**
Lopez (1992) [33]	0	3	3	0	0	1	1	0	-	-	1	0	0	0	0	0	**9/42 = 21%**
Marti & Sindelar (2015) [30]	3	3	3	0	1	3	2	3	2	3	-	3	3	-	0	1	**30/42 = 71%**

Market Research Document (1991) [41]	1	2	3	1	0	1	1	0	-	-	0	0	0	0	0	1	**10/42 = 24%**
Shai Balaban Dickinson Research Inc (n.d) [42]	1	2	3	2	0	1	1	0	-	-	0	1	1	0	0	0	**12/42 = 29%**
Stern D (1990) [45]	2	3	2	2	1	2	1	0	-	-	0	0	0	0	0	1	**14/42 = 33.3%**
Office of Tobacco Control(2006) [18]	2	2	3	3	3	1	0	0	0	3	-	3	0	0	0	2	**22/42 = 52%**
Warner PA Assoc (1990) [37]	1	3	3	2	0	1	1	1	-	-	1	0	0	0	0	1	**14/42/ = 33%**
Warner PA Assoc (1990) [37]	2	3	3	0	0	1	0	0	-	-	2	0	0	0	0	0	**11/42 = 26%**
Wilson et al. (1987) [21]	2	2	3	3	3	1	1	1	0	0	-	3	2	-	0	0	**21/42 = 50 %**
Wolf (1993) 34]	0	3	3	0	0	1	1	0	-	-	1	0	0	0	0	0	**9/42 = 21 %**
% of maximum possible score obtained for all papers	44.4	81.0	93.7	39.7	27.0	55.6	30.2	17.5	28.6	66.7	23.8	27.0	20.6	0.0	1.6	15.9	

References

1. World Health Organization. Global Health Observatory (GHO) Data: Tobacco Control: Prevalence of Tobacco Smoking. Available online: http://www.who.int/gho/tobacco/use/en/ (accessed on 2 May 2022).
2. Organization, W.H. Tobacco Free Initiative (TFI): WHO Global Report on Trends in Tobacco Smoking 2000–2025—First Edition. Available online: https://www.who.int/tobacco/publications/surveillance/reportontrendstobaccosmoking/en/ (accessed on 3 July 2022).
3. Hecht, S.S.; Hatsukami, D.K. Smokeless tobacco and cigarette smoking: Chemical mechanisms and cancer prevention. *Nat. Rev. Cancer* **2022**, *22*, 143–155. https://doi.org/10.1038/s41568-021-00423-4.
4. World Health Organization. Tobacco. Available online: https://www.who.int/news-room/fact-sheets/detail/tobacco (accessed on 21 August 2021).
5. Singh, S.; Singh, J.; Biradar, B.; Sonam, M.; Chandra, S.; Samadi, F. Evaluation of salivary oxidative stress in oral lichen planus using malonaldehyde. *J. Oral Maxillofac. Pathol.* **2017**, *26*, 26–30. https://doi.org/10.4103/jomfp.jomfp_333_21.

6. Dewhirst, T. Package size matters: Tobacco packaging, retail merchandising and its influence on trial and impulse sales. *Tob. Control* **2018**, *27*, 600–602. https://doi.org/10.1136/tobaccocontrol-2017-053962.
7. U. S. Department of Health Human Services. Reducing Tobacco Use: A Report of the Surgeon General. Available online: https://health.gov/healthypeople/tools-action/browse-evidence-based-resources/reducing-tobacco-use-report-surgeon-general (accessed on 2 February 2022).
8. Tobacco Industry Watch: Southeast Asia Tobacco Control Alliance. Malaysia: TI plays Word Game to Roll Back Ban on Kiddie Packs. Available online: https://tobaccowatch.seatca.org/index.php/2017/09/10/malaysia-ti-plays-word-game-to-roll-back-ban-on-kiddie-packs/ (accessed on 4 April 2022).
9. Tobacco, B.A. The Sensible Regulation of Tobacco-British American Tobacco's Views. Available online: http://legacy.library.ucsf.edu/tid/zye51a99/pdf (accessed on 3 March 2020).
10. Lee, I.; Blackwell, A.K.M.; Scollo, M.; De-Loyde, K.; Morris, R.W.; Pilling, M.A.; Hollands, G.J.; Wakefield, M.; Munafò, M.R.; Marteau, T.M. Cigarette pack size and consumption: An adaptive randomised controlled trial. *BMC Public Health* **2021**, *21*, 1420. https://doi.org/10.1186/s12889-021-11413-4.
11. Chaloupka, F.; Warner, K.E. The economics of smoking. In *Handbook of Health Economics*; Culyer, A.J., Newhouse, J.P., Eds.; Elsevier: Amsterdam, The Netherlands, 2000; Volume 1, pp. 1539–1627.
12. Jha, P.; Chaloupka, F.J. *Tobacco Control in Developing Countries*; Oxford University Press: Oxford, UK, 2000.
13. World Health Organization. *WHO Report on the Global Tobacco Epidemic, 2008: The MPOWER Package*; WHO: Geneva, Switzerland, 2008.
14. World Health Organization, Region Office for South-East Asia. Global Youth Tobacco Survey (GYTS): Indonesia Report, 2014. Available online: https://apps.who.int/iris/handle/10665/205148 (accessed on 20 August 2021).
15. Astuti, P.A.S.; Freeman, B. Protecting Young Indonesian Hearts from Tobacco. Available online: https://theconversation.com/protecting-young-indonesian-hearts-from-tobacco-97554 (accessed on 19 August 2021).
16. Southeast Asia Tobacco Control Alliance. A Snapshot of the Tobacco Industry in ASEAN Region Bangkok: Southeast Asia Tobacco Control. Available online: https://seatca.org/dmdocuments/TI%20Snapshot%202019.pdf (accessed on 6 July 2021).
17. Southeast Asia Tobacco Control Alliance. Keep the Ban on 'Kiddie Packs' to Save our Children—National Cancer Society Malaysia. Available online: https://seatca.org/keep-the-ban-on-kiddie-packs-to-save-our-children-national-cancer-society-malaysia/ (accessed on 20 August 2021).
18. Office of Tobacco Control. *Children, Youth and Tobacco: Behaviour, Perceptions and Public Attitudes*; Office of Tobacco Control: Naas, Ireland, 2006.
19. World Health Organization. WHO Framework Convention on Tobacco Control. Available online: http://apps.who.int/iris/bitstream/handle/10665/42811/9241591013.pdf?sequence=1 (accessed on 1 January 2022).
20. Lal, P.; Kumar, R.; Ray, S.; Sharma, N.; Bhattarcharya, B.; Mishra, D.; Sinha, M.K.; Christian, A.; Rathinam, A.; Singh, G. The Single Cigarette Economy in India—A Back of the Envelope Survey to Estimate its Magnitude. *Asian Pac. J. Cancer Prev. APJCP* **2015**, *16*, 5579–5582. https://doi.org/10.7314/apjcp.2015.16.13.5579.
21. Wilson, D.H.; Wakefield, M.A.; Esterman, A.; Baker, C.C. 15's: They fit in everywhere--especially the school bag: A survey of purchases of packets of 15 cigarettes by 14 and 15 year olds in South Australia. *Community Health Stud.* **1987**, *11*, 16s–20s. https://doi.org/10.1111/j.1753-6405.1987.tb00508.x.
22. Cunningham, R. Smoke and Mirrors. Available online: http://hdl.handle.net/10625/14226 (accessed on 10 March 2021).
23. Tan, Y.; Dorotheo, U. The Tobacco Control Atlas: ASEAN Region. Available online: https://aseantobaccocontrolatlas.org/ (accessed on 20 November 2021).
24. Bernama. *No To Kiddie Pack Cigarettes—Dr Subramaniam*; Bernama: Federal Territory of Kuala Lumpur, Malaysia, 2018.
25. Hollands, G.J.; Shemilt, I.; Marteau, T.M.; Jebb, S.A.; Lewis, H.B.; Wei, Y.; Higgins, J.; Ogilvie, D. Portion, package or tableware size for changing selection and consumption of food, alcohol and tobacco. *Cochrane Database Syst. Rev.* **2015**, *2017*, CD011045. https://doi.org/10.1002/14651858.CD011045.pub2.
26. Persoskie, A.; Donaldson, E.A.; Ryant, C. How tobacco companies have used package quantity for consumer targeting. *Tob. Control* **2018**, *28*, 365–373. https://doi.org/10.1136/tobaccocontrol-2017-053993.
27. Mat Rifin, H.; Rodzlan Hasani, W.S.; Ling, M.Y.J.; Robert Lourdes, T.G.; Saminathan, T.A.; Ab Majid, N.L.; Ahmad, A.; Ismail, H.; Mohd Yusoff, M.F. A systematic review protocol on small/kiddie cigarette packaging size and its impact on smoking. *Syst. Rev.* **2020**, *9*, 13. https://doi.org/10.1186/s13643-019-1263-6.
28. Gomez, M.L.; Guevara, M. Consumer Research Report. 1993. Available online: https://www.industrydocuments.ucsf.edu/docs/rtpy0013 (accessed on 28 November 2021).

29. Blackwell, A.K.M.; Lee, I.; Scollo, M.; Wakefield, M.; Munafo, M.R.; Marteau, T.M. Should cigarette pack sizes be capped? *Addiction* **2020**, *115*, 802–809. https://doi.org/10.1111/add.14770.
30. Marti, J.; Sindelar, J. Smaller Cigarette Pack as a Commitment to Smoke Less? Insights from Behavioral Economics. *PLoS ONE* **2015**, *10*, e0137520. https://doi.org/10.1371/journal.pone.0137520.
31. Farrell, L.; Fry, T.R.L.; Harris, M.N. 'A pack a day for 20 years': Smoking and cigarette pack sizes. *Appl. Econ.* **2011**, *43*, 2833–2842. https://doi.org/10.1080/00036840903389838.
32. Levy, J., Wood, D. *10's in-Depth Interviews-950817*; Philip Morris: New York, NY, USA, 1995.
33. Lopez, A. Virginia Slims Promotion and Advertising Study—Flash Report. 1992. Available online: https://www.industrydocuments.ucsf.edu/docs/lybn0105 (accessed on 10 March 2022).
34. Wolf, M. Special 10's Qualitative Research; 1993. Available online: https://www.industrydocuments.ucsf.edu/docs/frbl0122 (accessed on 10 March 2022).
35. Curtis, J. Alternative Pack Concepts for Kim, a New Female-Oriented Cigarette—A Unifocus Study. 1985. Available online: https://www.industrydocuments.ucsf.edu/docs/qxkd0132 (accessed on 10 March 2022).
36. Carter, S. Summary Analysis of Eight Focus Discussion Groups on Cigarette 12 Pack for Lorillard (861100). Available online: https://www.industrydocuments.ucsf.edu/docs/#id=xnll0115 (accessed on 28 November 2021).
37. Paul, A. Warner Associates Management Summary on Reactions to New Creative, Packaging and Promotions for Salem and Salem Box. 1990. Available online: https://www.industrydocuments.ucsf.edu/docs/#id=zzhg0059 (accessed on 28 November 2021).
38. Gomez, M.L. Consumer Research Report. In Islandwide Tracking Study May 1996 (19960500). Consumerresearch report. Mpr no. pr003/96. Islandwide Tracking Study Final Report. 1996. Available online: https://www.industrydocumentslibrary.ucsf.edu/tobacco/docs/qjwv0187 (accessed on 10 March 2022).
39. Burke Marketing Research. *Burke Marketing Research Package Size Evaluation Study*; Burke, Inc.: Cincinnati, OH, USA, 1983.
40. Generation Idea. Semi-Rigid Package Study. A Qualitative Exploration of Consumer Reactions to a New Type of Packaging for Cigarettes; Burke, Inc.: Cincinnati, OH, USA, 1986.
41. Market Research Document. *Segmentation—Phase I—Focus Group Research—Ontario/Quebec*; Burke, Inc.: Cincinnati, OH, USA, 1991.
42. Shoi Balaban Dickinson Research Inc. *An Exploratory Study—Pricing: Price-Conscious vs. Non-Price conscious Smokers*; Shoi Balaban Dickinson Research Inc.: Ft. Lauderdale, FL, USA, 1983.
43. Cox, A. Twelve Pack Focus Groups. 1983. Available online: https://www.industrydocuments.ucsf.edu/docs/styp0094 (accessed on 28 November 2021).
44. Ellison Quarterly Research. A Qualitative Analysis. Camel New Product Concept Screening Exploration. 1991. Available online: https://www.industrydocuments.ucsf.edu/docs/fnvp0081 (accessed on 28 November 2021).
45. Stern, D. Cartier in-Store Test. 1990. Available online: https://www.industrydocuments.ucsf.edu/docs/pkkk0129 (accessed on 28 November 2021).
46. Causey, R. Kent Qualitative Research—Paraguay. 1982. Available online: https://indusrtydocuments.library.ucsf.edu/tobacco/docs/ftcf01146 (accessed on 28 November 2021).
47. Sirriyeh, R.; Lawton, R.; Gardner, P.; Armitage, G. Reviewing studies with diverse designs: The development and evaluation of a new tool. *J. Eval. Clin. Pract.* **2012**, *18*, 746–752. https://doi.org/10.1111/j.1365-2753.2011.01662.x.
48. Vyth, E.L.; Steenhuis, I.H.; Brandt, H.E.; Roodenburg, A.J.; Brug, J.; Seidell, J.C. Methodological quality of front-of-pack labeling studies: A review plus identification of research challenges. *Nutr. Rev.* **2012**, *70*, 709–720. https://doi.org/10.1111/j.1753-4887.2012.00535.x.
49. Hughes, N.; Arora, M.; Grills, N. Perceptions and impact of plain packaging of tobacco products in low and middle income countries, middle to upper income countries and low-income settings in high-income countries: A systematic review of the literature. *BMJ Open* **2016**, *6*, e010391. https://doi.org/10.1136/bmjopen-2015-010391.
50. Adam, A.; Jensen, J.D. What is the effectiveness of obesity related interventions at retail grocery stores and supermarkets? -a systematic review. *BMC Public Health* **2016**, *16*, 1247. https://doi.org/10.1186/s12889-016-3985-x.
51. Finch, K.; Lawrence, D.; Williams, M.O.; Thompson, A.R.; Hartwright, C. A Systematic Review of the Effectiveness of Safewards: Has Enthusiasm Exceeded Evidence? *Issues Ment. Health Nurs.* **2022**, *43*, 119–136. https://doi.org/10.1080/01612840.2021.1967533.
52. Paul, A. Warner Associates. Management Summary of Findings on New Menthol Brand Packaging, Premiums and Promotional Concept. 1990. Available online: https://www.industrydocuments.ucsf.edu/docs//zzhg0059 (accessed on 28 November 2021).

53. Singh, M.D.V.; Kumar, R.; Kumar, A.M. ‘Loose’ cigarettes association with intensity of smoking: A secondary data analysis from Global Adult Tobacco survey, India 2009–2010. *J. Sci. Soc.* **2017**, *44*, 26–30.
54. Dangol, G.; Poudel, K.C.; Kim-Mozeleski, J.E. The Role of Parental Involvement in Cigarette Smoking among Adolescents in Nepal. *J. Psychoact. Drugs* **2022**, *54*, 110–118. https://doi.org/10.1080/02791072.2021.1923875.
55. Ekpu, V.U.; Brown, A.K. The Economic Impact of Smoking and of Reducing Smoking Prevalence: Review of Evidence. *Tob. Use Insights* **2015**, *8*, 1–35. https://doi.org/10.4137/tui.S15628.
56. National Center for Chronic Disease Prevention and Health Promotion (US) Office on Smoking and Health. 5, The Tobacco Industry’s Influences on the Use of Tobacco Among Youth. In *Preventing Tobacco Use Among Youth and Young Adults: A Report of the Surgeon General*; Centers for Disease Control and Prevention (US): Atlanta, GA, USA, 2012.
57. Department of Psychology, University of Waterloo. *International Tobacco Control (ITC) Policy Evaluation Project: ITC Malaysia Wave 1–4 National Report (2005–2009)*; University of Waterloo: Waterloo, Canada, 2012.
58. van Schalkwyk, M.C.I.; McKee, M.; Been, J.V.; Millett, C.; Filippidis, F.T. Size matters: An analysis of cigarette pack sizes across 23 European Union countries using Euromonitor data, 2006 to 2017. *PLoS ONE* **2020**, *15*, e0237513. https://doi.org/10.1371/journal.pone.0237513.
59. Page, M.J.; McKenzie, J.E.; Bossuyt, P.M.; Boutron, I.; Hoffmann, T.C.; Mulrow, C.D.; Shamseer, L.; Tetzlaff, J.M.; Akl, E.A.; Brennan, S.E.; et al. The PRISMA 2020 statement: an updated guideline for reporting systematic reviews. *BMJ* **2021**, *372*, n71. https://doi.org/10.1136/bmj.n71.
